# Translating a Preclinical Hydrogel Platform into a Human Therapeutic for Delivering Targeted Low-Dose Anti-CTLA-4

**DOI:** 10.3390/gels12060489

**Published:** 2026-06-02

**Authors:** Airi Harui, Michael D. Roth

**Affiliations:** Division of Pulmonary & Critical Care, Department of Medicine, David Geffen School of Medicine at UCLA, Los Angeles, CA 90095-1690, USA; aharui@mednet.ucla.edu

**Keywords:** checkpoint inhibitor, cytotoxic T-lymphocyte-associated antigen 4 (CTLA-4), ipilimumab, cancer immunotherapy, hydrogel, hyaluronic acid, recombinant human hyaluronidase (rHuPH20), shear storage modulus, cryogenic scanning electron microscopy, low-dose

## Abstract

Systemic administration of antibodies that target immune checkpoint inhibitor pathways is a highly effective approach to cancer immunotherapy, but systemic toxicity can limit clinical utility. In preclinical testing, a peri-tumor injection of a low dose of hydrogel-encapsulated cytotoxic T-lymphocyte-associated antigen 4 (CTLA-4) antibody was shown to selectively activate T cells in tumor-draining lymph nodes, induce tumor infiltration by cytotoxic T cells, and result in tumor regression, protective immunity, and long-term survival. In contrast to systemic therapy, there was limited systemic exposure or risk for autoimmune toxicity. The current study focuses on translating this platform into a biocompatible human therapeutic. The hydrogel matrix was reformulated using a low-molecular-weight hyaluronic acid. A recombinant human hyaluronidase (rHuPH20) was incorporated to promote lymph node targeting and self-resorbing features. Formulations were optimized to operate at neutral pH and with gelation kinetics allowing a 5 to 10 min administration window. Performance features were assessed including the capacity to encapsulate human IgG or ipilimumab antibody at proposed therapeutic doses (1–15 mg/mL), impact of rHuPH20 and antibody on rheologic properties and three-dimensional microstructure, and payload delivery profiles in vitro and in vivo. Results confirm the capacity for this unique hydrogel platform to be adapted for human testing.

## 1. Introduction

Immune checkpoint inhibitors (ICIs) play an essential role in preventing unwanted reactions to self-antigens (autoimmunity) and limiting the magnitude of immune responses [1,2]. Cancers that co-opt ICI pathways can therefore suppress the activation of tumor-reactive T cells, expand the production of regulatory T cells (Tregs) at the expense of effector T cells, and promote T cell exhaustion [3]. It is in this setting that the development of monoclonal antibodies (mAbs) that block cytotoxic T-lymphocyte antigen 4 (CTLA-4), programmed cell death protein 1 (PD-1) and programmed death-ligand 1 (PD-L1), have emerged as highly effective treatments for cancer [4,5]. The most potent cancer responses are observed when patients are treated with infusions of mAbs targeting CTLA-4 along with either PD-1 or PD-L1. The resulting “combination therapies” benefit from a synergistic interaction that dramatically increases cancer response rates and overall survival [3,4,6]. Unfortunately, combination therapies also dramatically increase the frequency and severity of immune-related adverse events (irAEs) that can limit tolerability and overall utility [7,8]. As irAEs are dose-dependent and primarily occur in organ systems unrelated to the primary tumor, there is a growing interest in low-dose therapies that target the tumor microenvironment and tumor-draining lymph nodes (TDLNs) while limiting systemic exposure [9,10].

The primary suppressive effects from tumor-mediated CTLA-4 signaling occur at the level of TDLN [3,4]. Within TDLN, antigen-presenting cells would normally initiate the activation of tumor-reactive T cells and stimulate the clonal expansion and systemic release of effector T cells. In some of the earliest investigations into targeted therapy, tumor cell lines were gene-modified to secrete a functional anti-CTLA-4 mAb [11]. Continuous local release of anti-CTLA-4 at the site of implanted tumor cells was associated with delayed tumor growth and a reduction in the generation of Tregs. When combined with another systemic therapy to reduce Tregs, tumors completely regressed, animals developed immunity and the irAEs normally associated with systemic anti-CTLA-4 were not observed. Fransen et al. [12] later demonstrated that immune-mediated tumor killing and improved survival could be obtained in tumor-bearing mice by injecting low-dose anti-CTLA-4, emulsified within a slow-release adjuvant, in the distribution of TDLN but not elsewhere. Our own research demonstrated significantly greater potency when anti-CTLA-4 was encapsulated within an injectable hyaluronic acid (HA) hydrogel that was designed to release mAbs over 3–5 days at a peri-tumor location [13,14]. This treatment was far superior to the same dose of anti-CTLA-4, when given alone as a peri-tumor injection, and even outperformed high-dose systemic therapy. Serum anti-CTLA-4 levels produced by the hydrogel release were minimal when compared to systemic dosing and there was minimal activation of autoimmunity. More importantly, hydrogel-based dosing in combination with systemic administration of an anti-PD-1 antibody produced a superior and synergistic response; replicating the effects of conventional combination therapy. Others have also documented that the efficacy of low-dose targeted anti-CTLA-4, alone or in combination with anti-PD-1, is enhanced when using a controlled-release delivery mechanism and occurs without associated toxicity [15,16,17]. As a proof of concept, injection of low doses of anti-CTLA-4 antibody in the distribution of a TDLN led to a significant reduction in the expression of Tregs and enhancement in the frequency of effector T cells within both the TDLN and systemic circulation of melanoma patients [18].

In order to prepare our hydrogel-encapsulated anti-CTLA platform for clinical testing, the research presented here explores the shift in formulation priorities required for the transition to a clinical-grade product, identifies reagents and formulations meeting these specifications, and examines the impact of anticipated human antibody dosing and human hyaluronidase on rheology, three-dimensional hydrogel architecture, antibody release profile in vitro and serum pharmacokinetics (PK) in a murine model.

## 2. Results and Discussion

### 2.1. Transition to a Clinical-Grade Hydrogel Composition

For pre-clinical testing, a unique hydrogel reagent was specifically developed as an injectable tissue reservoir intended to perfuse TDLN with low-dose anti-CTLA-4 for 3–5 days [13,14]. Key structural components included a commercial research-grade thiolated carboxymethyl HA, as a natural polymer, and poly-(ethylene glycol)-diacrylate (PEG-DA), as a crosslinker that initiates spontaneous polymerization via a Michael addition reaction between the thiol and acrylate groups [19,20]. The concentrations and ratios for these components were modified from those of typical commercial hydrogels as a means to specifically control the release of therapeutic mAbs. Anti-mouse CTLA-4 was encapsulated as the payload and a purified bovine hyaluronidase incorporated to modulate both the hydrogel structure and the surrounding tissue environment. The addition of hyaluronidase significantly enhanced lymphatic uptake and targeting of anti-CTLA-4 mAb to TDLN, resulting in a substantial improvement in the treatment response [14]. Translating this into a viable clinical product required a shift in formulation priorities [Table 1]. In addition to the shift from research reagents to those compatible with current good manufacturing practices (cGMP), a clinical hydrogel formulation needs to assure human immunologic and biologic compatibility, consider the intended sites for human administration, and address anticipated human dose ranges, dosing schedules and required drug release profiles [21].

The primary matrix constituents, HA and PEG-DA, are consistent with other hydrogel formulations that have been extensively tested in humans and are considered biocompatible for soft-tissue injection [19,20,21,22,23]. The molecular weight of the HA component impacts solubility, rheologic features, immunologic effects and other aspects of intended performance [24,25,26]. The pre-clinical HA reagent, manufactured with a broader range of molecular weights, was replaced with a purified 100 kDa formulation primarily due to its superior solubility. The higher solubility facilitated the use of higher concentrations and simplified reconstitution at the point of use. In the process of translating our hydrogel platform for clinical use, over 70 different formulations were evaluated as part of an iterative process to identify a mixture of HA and PEG-DA (1.2% *w*/*v* final for HA; 1.0% *w*/*v* final for PEG-DA) that met all of the desired performance features [Table 1]. This formulation is the focus of the research presented here. Crosslinked HA hydrogel formulations typically remain as stable space-occupying fillers at the site of injection for 6–24 months [20,27]. In this application, where repeated cycles of anti-CTLA-4 will need to be administered at the same site, a hyaluronidase enzyme was incorporated to create a self-degrading feature [14,24]. The original bovine hyaluronidase was replaced by a biosimilar for the FDA-approved human hyaluronidase-1 (rHuPH20). Recombinant HuPH20 is used clinically to facilitate the administration of large volume subcutaneous injections and the rapid transfer of antibody payloads to the bloodstream [28,29]. In such applications, rHuPH20 produces a temporary breakdown of HA within the subcutaneous tissue, at the site of injection, to facilitate uptake and systemic dosing. Recombinant HuPH20 can also be injected directly into existing HA-based cosmetic fillers as a mechanism to promote their rapid resorption [30]. In the clinical reagent proposed here, rHuPH20 plays multiple roles to: (1) control hydrogel rheology; (2) promote time-controlled release of encapsulated antibody; (3) facilitate matrix resorption to allow repeated administration at the same site; (4) enhance lymphatic uptake and thereby target released anti-CTLA-4 directly to TDLN [14]; and (5) generate low-molecular-weight HA fragments with potential immuno-stimulatory effects [13,14,25]. Finally, the low concentrations of murine antibodies that were incorporated into our preclinical hydrogels (up to 330 µg/mL) were replaced with either clinical-grade human immunoglobulin G (IgG) or a mAb biosimilar for ipilimumab (human anti-CTLA-4) at anticipated therapeutic concentrations ranging from 1 to 15 mg/mL [18].

### 2.2. Gelation and Rheological Properties

Gelation kinetics, mechanical stiffness and the deformation elasticity of hydrogels are critical parameters that drive their utility for different applications. For the indications proposed here, targeted goals included: (1) adequate gelation time to allow loading and injection at a targeted peri-tumor location before polymerization impairs flow through the needle (5–10 min) and (2) a matrix stiffness compatible with that of intended soft-tissue injection sites. Cancers for which anti-CTLA-4 therapy is already approved range from locations within/adjacent to compliant soft tissues (subcutaneous tissue, lung, and esophageal/bowel wall) to more solid organs such as the liver and kidney. The shear storage moduli (G′) in these various tissues usually range from 0.1 to 10 kPa, with most under 3 kPa [31]. Reports on the G′ for several commercial HA-based dermal fillers document a range from 40 to 1000 Pa, with the differences in stiffness related to the intended use for subtle cosmetic changes versus volumizing and lifting [23]. Assessing the change in G′ over time also allowed us to assess the time from mixing to the onset of rapid gelation (working time) and the stability of the hydrogel matrix over time when formulated with rHuPH20.

**Figure 1:**
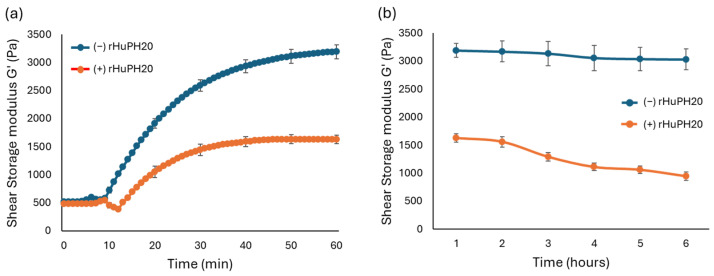
Effect of recombinant human hyaluronidase-1 (rHuPH20) on the hydrogel shear storage modulus (G′; Pa) during initial polymerization and after attaining maximal stiffness. Hyaluronic acid (HA; 1.2% *w*/*v* final), poly-(ethylene glycol)-diacrylate (PEG-DA; 1.0% *w*/*v* final), and human immunoglobulin G (IgG; 10 mg/mL final) are mixed, in the presence or absence of rHuPH20 (1660 IU/mL final), and vortexed to initiate gelation. The hydrogel mixtures are immediately placed into the rheometer and monitored over time for G′ (Pa) under simulated tissue conditions (37 °C, humidified). (**a**) Time course of the change in G′ during the initial polymerization phase (1–60 min). Graphs display the mean (±SE) from 4 experiments. (**b**) Stability of G′ after attaining maximal stiffness, 1 to 6 h after initiating polymerization. Graphs display the mean (±SE) from 3 experiments.

We first evaluated the impact of rHuPH20 (1660 IU/mL) on the kinetics of G′ during the initial 60 min of polymerization and gelation. As shown in Figure 1a, the transition from a liquid to solid phase occurred abruptly between 8 and 12 min after mixing. In clinical practice, this provides adequate time for preparing the injection and confirming final needle placement. Inclusion of rHuPH20 prolonged the time to onset of gelation by a few minutes, reduced the rate of rise in G′ and the ultimate hydrogel stiffness. At 60 min, when G′ had reached a plateau for both conditions, the shear storage modulus was 3190 ± 125 Pa (mean ± SE) for the hydrogel formulation that lacked rHuPH20, but only 1625 ± 75 Pa when rHuPH20 was included (a 49% reduction in G′). A shear storage modulus in this range is anticipated to be compatible with most soft-tissue sites [31].

When rheologic characteristics were evaluated over a longer time (6 h), the basic hydrogel matrix remained very stable, consistent with the long-term stability of conventional HA hydrogels (Figure 1b). However, the addition of rHuPH20 induced a progressive and significant (*p* = 0.007) decrease in G′ from 1630 ± 106 Pa at 1 h after the start of polymerization to 944 ± 84 Pa at 6 h, representing a further 42% reduction in stiffness. The closer that a tissue matches with the inherent stiffness and elasticity of the target tissue, the less impact it has on local tissue fluid dynamics and the less impact that the tissue has on compression and spreading of the hydrogel matrix. The self-resorbing nature of this formulation should also reduce the risk of pressure-associated complications.

In our hands, gelation behavior and stiffness were not impacted by loading the hydrogel with antibody concentrations in the range of 1 to 15 mg/mL (Supplementary Figure S1). However, at 20 mg/mL, the shear storage modulus could not be measured, suggesting that a maximum antibody concentration compatible with hydrogel polymerization lies between 15 and 20 mg/mL for this formulation.

### 2.3. Cryogenic Scanning Electron Microscopy (Cryo-SEM) Analysis of Hydrogel Structures

**Figure 2:**
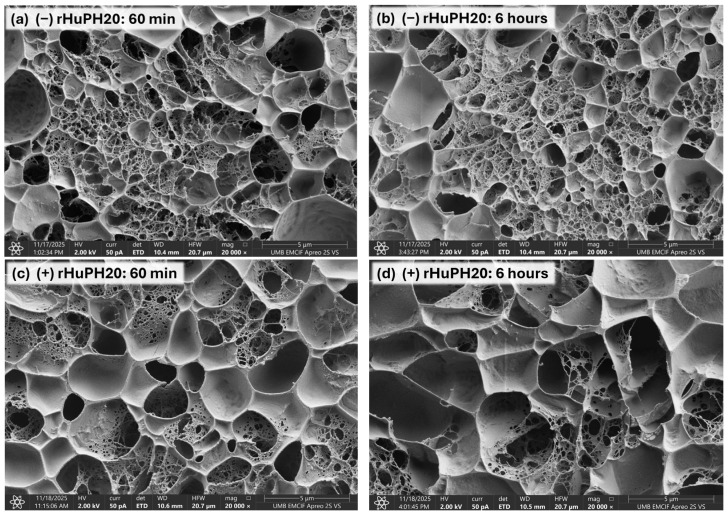
Impact of time and rHuPH20 on three-dimensional hydrogel structure as assessed by cryogenic scanning electron microscopy (Cryo-SEM). Hyaluronic acid (1.2% *w*/*v* final), PEG-DA (1.0% *w*/*v* final), and human IgG (10 mg/mL final) are mixed, in the presence or absence of rHuPH20 (1660 IU/mL), cultured at 37 °C, and flash-frozen in liquid nitrogen at 60 min (**a**,**c**) or 6 h (**b**,**d**) for subsequent imaging by Cryo-SEM (20K magnification). The effects of rHuPH20 on structure are demonstrated by comparing hydrogels that lack rHuPH20 (**a**,**b**) to those containing rHuPH20 (**c**,**d**). Representative images from 3 experiments.

To gain insight into the three-dimensional architecture of our polymerized hydrogels, we examined their microstructure by Cryo-SEM (Figure 2). In the absence of rHuPH20 (Figure 2a,b), the underlying hydrogel formulation at 60 min exhibited a heterogeneous porous structure with a mean diameter (±SD) of 1.05 (±0.70) µm (range 0.03 to 4.35 µm). The walls of each pore were composed of a fishnet of microporous structures with a much smaller mean diameter (±SD) of 118 (±51) nm (range 9 to 325 nm). These structural features remained unchanged at 6 h after gelation, consistent with the stability observed over the same interval when evaluated by rheology.

While the overall three-dimensional structure appeared similar when hydrogels were formulated in the presence of rHuPH20, the pore sizes were obviously larger and there were progressive changes in the architecture over time. At 6 h, the mean diameter (±SD) of the pores increased to 3.73 (±1.57) µm (range 1.42 to 8.22 µm). In addition, there were obvious areas where the fishnet of microporous structures making up the side walls appeared severed and/or the diameters enlarged. As a result, their mean diameter (±SD) increased to 279 (±189) nm (range 66 to 1082 nm), consistent with a progressive enzymatic destruction.

It is well established that the kinetics of protein retention and release for this class of HA hydrogels are dependent on several features including protein size, HA methylation rate, the concentrations and molecular weights of both HA and PEG-DA, and their ratios [19,20,32,33]. Given that the length and width of a human IgG molecule is in the range of 8–10 nm [34], our Cryo-SEM findings suggest that the observed pore structures, with diameters in the µm range, act as the primary IgG reservoirs while the microporous composition of their walls, with pore diameters in the nm ranges, retard the mass transit of antibodies and control IgG release kinetics. In our formulation, the addition of rHuPH20 impacts both the pore diameters and the microporous structures that make up their walls. As a result, the hydrogel matrix containing rHuPH20 is softer (smaller G′), the pores are larger, and with time the encapsulated IgG is released at a progressively faster rate as the matrix is degraded and the gel softens further.

### 2.4. Capacity for Loading and Releasing Human IgG

**Figure 3:**
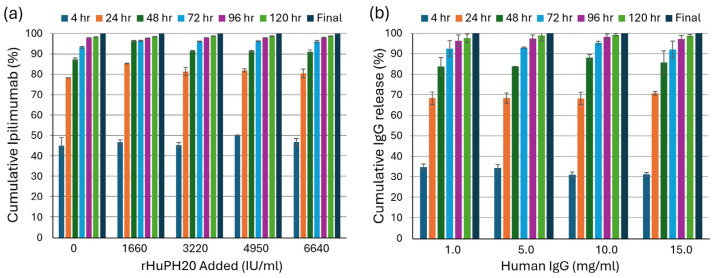
Hydrogel in vitro release characteristics for ipilimumab and human IgG. Hydrogels are prepared in tubes with either: (**a**) ipilimumab (human anti-CTLA-4) at 1 mg/mL along with varying concentrations of rHuPH20 (0–6640 IU/mL) (representative experiment with means ± SD for duplicate measures shown, *n* = 2 experiments) or (**b**) human IgG at concentrations from 1 to 15 mg/mL along with rHuPH20 at 1660 IU/mL (representative experiment with means ± SD for duplicate measures shown, *n* = 3 experiments) and examined for antibody release into 1 mL of media (at 37 °C) at defined time points. Media is completely removed and replaced with fresh media at each time point, with bovine hyaluronidase (1000 IU) added after 120 h to promote complete lysis prior to the “Final” measurement. Recovery at each time point is normalized as a percentage of the total antibody recovered. Measurements by antigen-specific ELISA.

Having reformulated several aspects of this injectable hydrogel platform to prepare it for clinical applications (humanization), we next evaluated its capacity to encapsulate and release human IgG and anti-human CTLA-4. In initial testing, a biosimilar for ipilimumab was incorporated into our hydrogel formulation at a concentration of 1 mg/mL and examined for its release characteristics in vitro across a range of rHuPH20 dosing (1600–6640 IU/mL) commonly employed for subcutaneous injections [29,35]. A release media was added to each hydrogel 20 min after initiating polymerization and samples incubated at 37 °C. At described intervals, the release media was completely recovered for antibody measurement and replaced with fresh media. At the end of 5 days, 1000 IU of bovine hyaluronidase was added to dissolve any remaining hydrogel matrix prior to a final collection. The time course for release of ipilimumab was plotted (Figure 3a). An initial bolus release, amounting to between 35 and 50% of the loaded antibody, occurred within the first 4 h, regardless of the presence or absence of rHuPH20, and approximately 98% of the antibody was released by the end of 5 days, closely resembling the anti-mouse CTLA-4 release profile reported previously [13,14]. Documenting complete release of antibody from the hydrogel matrix within a specified timeframe is a critical outcome from a regulatory perspective. The presence of rHuPH20 had no significant impact on these key outcomes, but the presence of rHuPH20 at any of these doses (1600–6640 IU/mL) was associated with a slight increase in fractional release of ipilimumab at the 24- and 48 h timepoints. As such, the addition of rHuPH20 concentrations in the range of 1600 to 6400 IU/mL does not interfere with the clinical goal of an initial bolus followed by an ongoing release of therapeutic antibody over a period of 3–5 days.

Rheology studies suggested no significant impact of human IgG concentration on gelation or extended rheology kinetics. We therefore formulated hydrogels across the intended therapeutic range of 1–15 mg/mL and examined their release kinetics in the presence of 1660 IU rHuPH20 (Figure 3b). All formulations, regardless of the dose of IgG loaded, exhibited similar release profiles without significant differences between groups. In this setting, the current formulation appears stable with respect to antibody loading and release within this range. As such, a single formulation of the underlying hydrogel matrix can be used for dose–response testing of ipilimumab in future clinical investigations.

### 2.5. Serum Exposure to Ipilimumab in an In Vivo Animal Model

**Figure 4:**
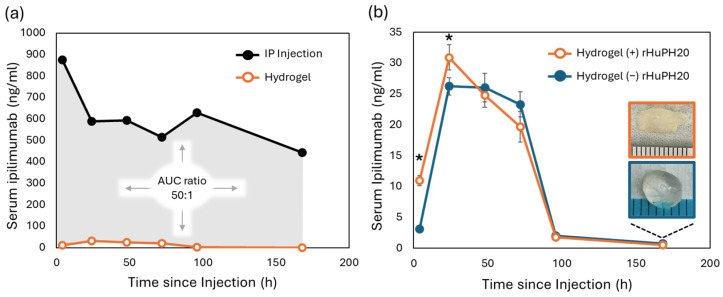
Serum pharmacokinetics (PK) following subcutaneous injection of low-dose hydrogel-encapsulated ipilimumab (0.15 mg) or a higher dose intraperitoneal (IP) injection (1.5 mg) of ipilimumab. Immunodeficient non-obese diabetic mice, with severe combined immune deficiency and interleukin-2 receptor common gamma chain-deficient (NSG strain), are treated with either subcutaneous injection of hydrogel-encapsulated low-dose anti-CTLA-4 (0.15 mg/150 µL hydrogel) or IP injection of high-dose anti-CTLA-4 (1.5 mg in PBS). Serum is collected at 4-, 24-, 48-, 72-, 96- and 168 h after the injections and ipilimumab concentration is determined by ELISA. (**a**) Overall serum exposure, as measured by the area under the curve (AUC) for ipilimumab concentration, is 50 times greater when a high dose is administered by IP injection as compared to a low dose administered by hydrogel injection. (**b**) The transfer of ipilimumab from subcutaneous tissue to the serum compartment is greater at early timepoints when the hydrogel includes rHuPH20 (* *p* < 0.05), but there is no impact of rHuPH20 on the overall AUC exposure following hydrogel administration. At the end of 7 days, hydrogels are recovered for visual inspection (see representative image inserts). Data displayed as means ± SE, *N* = 5–6 mice/group. Representative experiments shown; *n* = 3 experiments.

In pre-clinical animal studies, the small doses of anti-CTLA-4 antibody delivered to TDLN by our hydrogel platform, when compared to much larger systemic dosing, were shown to significantly reduce systemic autoimmune toxicity while maintaining comparable (or better) anti-tumor efficacy [13,14]. This approach maximized exposure at TDLN while dramatically reducing peak serum antibody levels and overall area under the curve (AUC) exposure by >95%. To test whether this humanized hydrogel formulation would perform similarly, serum PK was carried out in non-obese diabetic mice with severe combined immune deficiency and deficient for the interleukin-2 receptor common gamma chain (NSG mice) that lack the capacity to mount immunologic responses to human antigens [36]. Mice received either a single subcutaneous injection of hydrogel containing ipilimumab (0.15 mg in 150 μL hydrogel) or an intraperitoneal (IP) injection of 1.5 mg of the same antibody in PBS to simulate the proposed 10-fold difference between anticipated human targeted versus systemic dosing (Figure 4).

As shown in Figure 4a, systemic administration resulted in a peak serum ipilimumab concentration of 876.2 ng/mL at 4 h, whereas the 10-fold lower dose of ipilimumab delivered by subcutaneous hydrogel injection (containing rHuPH20) resulted in a peak of only 30.9 ng/mL at 24 h. As such, there was a 24-fold difference in peak serum exposure, similar to prior results with mouse anti-CTLA-4 antibody [13,14]. The impact of hydrogel administration on AUC was even more striking. The 7-day AUC was approximately 50-fold lower when comparing the hydrogel-treated animals to those receiving a systemic injection (1963 ng/ml·h vs. 94,593 ng/ml·h).

To understand the role that rHuPH20 has on systemic exposure, we also compared the serum PK between hydrogels that were formulated with and without rHuPH20 (Figure 4b). Inclusion of rHuPH20 was associated with a slight increase in serum exposure at early timepoints (4 and 24 h), but there was no significant impact on the overall AUC (1963 ng/mL·h vs. 1920 ng/mL·h). When hydrogels were retrieved from the subcutaneous tissue of animals at the end of the 7 days, there was a clear difference in appearance. Hydrogels that were injected in the absence of human hyaluronidase were firmer, retained a symmetric spherical shape and were visually clear and translucent. In contrast, the inclusion of rHuPH20 resulted in recovered hydrogels that were very soft and gelatinous, irregularly shaped, and appeared highly infiltrated and opaque—often difficult to separate from the surrounding tissue. Collectively, animal modeling with the humanized formulation confirms that incorporation of lower doses of ipilimumab and administration at a soft-tissue site results in dramatically lower systemic exposure. In earlier animal testing, this marked reduction in systemic exposure correlated with a marked reduction in systemic autoimmune toxicity while still producing greater exposure at the level of TDLN and equal or better systemic tumor responses [13,14].

## 3. Conclusions

The development of a targeted anti-CTLA-4 therapeutic that will activate tumor-specific immune responses, while sparing the activation of systemic irAEs, could significantly enhance the tolerability and overall utility of ICI therapies [7,8,37]. In prior work, we established that an HA-based hydrogel could be modified to retain large proteins, the size of mAbs, and release them in a controlled manner over 3–5 days [32]. The inclusion of hyaluronidase facilitated antibody release, improved lymphatic uptake and lymph node perfusion, while providing a self-resorbing feature that facilitates repeated cycles of administration. In pre-clinical testing, a peri-tumor injection of this novel anti-CTLA-4 reagent activated T cells in TDLN, while sparing other lymph nodes, and led to systemic anti-cancer responses and immune memory that was more potent than high-dose systemic therapy or peri-tumor administration of anti-CTLA-4 alone [13,14]. Serum exposure and the induction of systemic autoimmune toxicity in mouse models were dramatically reduced.

The current studies focus on translating this platform into a formulation compatible with clinical application. A set of translational performance objectives were identified. Appropriate reagents for clinical-grade manufacturing were identified and the hydrogel formulation was modified to accommodate a low-molecular-weight HA to facilitate reconstitution at the point of use. A comprehensive analysis of the rheologic changes over time confirmed that the product provides adequate working time (5–10 min) to allow loading and needle injection. In addition, inclusion of rHuPH20 modified hydrogel stiffness within a range compatible with soft-tissue injection. Ongoing resorption of the HA matrix by rHuPH20 should prevent pressure-related complications at the site of injection and facilitate repeated injection at the same site. Cryo-SEM studies provide novel insight into the three-dimensional features of the hydrogel platform and document the progressive impact, over time, of included rHuPH20 on structure. Finally, in vitro release studies and PK testing in a murine model met predetermined performance standards. Collectively, these translational studies describe a hydrogel platform for the delivery of anti-CTLA-4 that can be readily adapted for clinical testing.

## 4. Materials and Methods

### 4.1. Reagents

A purified, low-endotoxin, anti-human CTLA-4 mAb, a biosimilar for ipilimumab [Clone MDX-010], was purchased in phosphate-buffered saline from Leinco Technologies, Inc. (St. Louis, MO, USA). GAMUNEX^®^-C Immune Globulin (Human) 10%, a clinical-grade therapeutic containing >98% human IgG, was purchased from McKesson (Irving, TX, USA). Hydrogel components, which included low molecular weight (100 kDa), animal-free, thiol-modified HA (Glycosil^®^, Carlsbad, CA, USA) and PEG-DA (Extralink^®^; Częstochowa, Poland, 3.4 kDa), were obtained from specified lots made available by Advanced BioMatrix, Inc. (Carlsbad, CA, USA). A biosimilar for an FDA-approved rHuPH20 was purchased from Creative Enzymes (Shirley, NY, USA). Purified bovine hyaluronidase used for ex vivo digestions was from MP Biomedicals (Santa Ana, CA, USA). ELISA kits detecting anti-human CTLA-4 and human IgG were purchased from Abcam (Waltham, MA, USA) and BioLegend (San Diego, CA, USA), respectively.

### 4.2. Hydrogel Formation

Self-polymerizing hydrogels were prepared as described previously [14]. Briefly, HA and PEG-DA were individually dissolved with respective buffers from a lyophilized state to prepare 2% and 10% (*w*/*v*) buffered solutions (pH 7.2–7.4), respectively. A mixture containing antibody (anti-human CTLA-4 or human IgG), rHuPH20 and PEG-DA was prepared and incubated for 10 min at room temperature before mixing with the thiolated HA solution to initiate gelation. Final concentrations of HA and PEG-DA were 1.2% and 1% (*w*/*v*), respectively, with the antibody payloads ranging from 1 to 15 mg/mL and rHuPH20 ranging from 0 to 6640 IU/mL.

### 4.3. Changes in Shear Storage Modulus (G′) over Time

Rheology testing was performed at Advanced Biomatrix, Inc., using a programmable ElastoSens™ Bio rheometer (Rheolution Inc., Montreal, QC, Canada). All hydrogel components were mixed, added to a 3 mL rheology test cup and immediately inserted into the rheometer. A humidified 37 °C test chamber was employed during testing to replicate body temperature and prevent dehydration of the hydrogel over time. Changes in G′ over time were recorded at 1 min intervals for the first 60 min and subsequently at 1 h intervals for up to 6 h.

### 4.4. Structural Microanalysis by Cryo-SEM

Cryo-SEM was performed at the University of Maryland, Baltimore, in the Electron Microscopy Core Imaging Facility. Hydrogels were incubated in a 37 °C water bath for specified time intervals after the initiation of gelation, then immediately cut into 2 mm × 4 mm pieces, mounted onto slotted stubs, and secured with Tissue-Tek^®^ O.C.T.™ cryo glue (Sakura Finetek USA, Inc., Torrance, CA, USA) mixed in equal proportion with Aquadag Colloidal Graphite ECO (Agar Scientific Ltd., Essex, UK). Mounted samples were plunge-frozen in pre-slushed liquid nitrogen using the Quorum PP3010T Cryo Preparation System (Quorum Technologies, East Sussex, UK) and either examined immediately or cryopreserved for subsequent analysis. After being placed onto a pre-cooled stage (−145 °C) in the cryogenic preparation chamber, the stage was warmed to −90 °C for freeze-fracture, followed by a 15 min sublimation to expose the hydrogel architecture. Following sublimation, the temperature of the preparation chamber was returned to −145 °C and a sputter-coater, integrated within the system, was used to coat with 5 nm platinum/palladium prior to placement onto the cryo stage (−145 °C) in a Thermo Fisher Scientific Apreo field-emission scanning electron microscope (Thermo Fisher Scientific, Brno, Czechia) for imaging. Cryo-SEM imaging was conducted at 2 kV accelerating voltage and 50 pA probe current using the Everhart–Thornley (ETD) secondary electron detector. All samples were imaged using identical acquisition settings, including a 10 mm working distance, 500 ns dwell time, and 16-frame averaging for accurate comparison across samples. Pore size distributions were performed using image editing in Adobe Photoshop and subsequent analysis of regions of interest using Image J.JS online software to identify the Feret’s diameter with a minimum of 100 pores measured for each analysis.

### 4.5. Antibody Payload Release Profiles In Vitro

In vitro assessments of hydrogel antibody release kinetics were performed as described previously [13,14]. Briefly, hydrogel solutions containing either anti-human CTLA-4 antibodies or human Ig were mixed in 4 mL tubes to initiate crosslinking and incubated at room temperature for 20 min to allow gelation. One ml of release medium (PBS with 1% BSA, 0.5 mM EDTA) was then added, followed by incubation at 37 °C. All of the release media was collected from each tube and replaced with fresh medium at 4, 24, 48, 72, 96, and 120 h. At the 120 h time point, 1 mL containing bovine hyaluronidase (1000 IU) was added to digest any remaining HA and release all remaining antibodies from the matrix. To quantify intact anti-human CTLA-4 antibodies with preserved binding activity, an ELISA specific for ipilimumab, which employed wells coated with human CTLA-4 antigen, was used. Total human IgG levels were quantified using a human IgG ELISA kit. All assays were performed according to the manufacturer’s protocols with replicate measurements.

### 4.6. Animals and In Vivo PK for the Release of Human Anti-CTLA-4

Immunodeficient NSG mice (strain NOD.Cg-Prkdc^scid^Il2rg^tm1Wjl^/SzJ, the Jackson Laboratory), which lack the capacity to mount xenograft responses, were bred and housed at the UCLA CFAR Humanized Mouse Core facility in individually ventilated cages with sterile food, water, and caging, and handled within Class II biosafety cabinets. All protocols and procedures were approved by the UCLA Animal Research Committee in accordance with all federal, state, and local guidelines.

Age (7–8 weeks old) and size-matched NSG mice were administered a single dose of human anti-CTLA-4 either by IP injection (1.5 mg) or by subcutaneous injection of 150 µL of a specified hydrogel formulation (containing 0.15 mg) administered to the loose tissue of the upper right back as previously detailed [13,14]. Serum samples were collected at 4-, 24-, 48-, 72-, 96- and 168 h post-injection. Dosing differences reflect anticipated differences between human therapeutic doses for systemic versus targeted hydrogel administration. Serum levels of human anti-CTLA-4 were quantified by ELISA as detailed above. Hydrogels were removed at the end of the experiment for assessment of their sizes and status.

### 4.7. Statistical Evaluation

The shear storage modulus G′ time course and the in vitro and in vivo hydrogel release profiles were compared between groups using ANOVA tests. *p* ≤ 0.05 was considered significant. Two-tailed *t*-tests were carried out for individual comparisons between two groups with *p* ≤ 0.05 considered significant. All testing was performed with replicate samples and repeated independent testing as detailed for each analysis.

## 5. Patents

Modified Hyaluronic Acid Hydrogels and Proteins for the Time-Controlled Release of Biologic Agents. US 10,842,743 (B2), Publication date 24 November 2020.

## Figures and Tables

**Table 1:** Formulation priorities for clinical translation of a pre-clinical hydrogel designed to deliver anti-CTLA-4 to tumor-draining lymph nodes (TDLNs).

Attributes	Pre-Clinical Formulation	Clinical Formulation
Reagent grades:	Research	cGMP/FDA-approved
Species-specificity:	Mouse/animal	Human
Injection site:	Subcutaneous tissue	Organ-specific soft tissue
Anti-CTLA-4 dose (mg):	0.05 ^1,2^	1–15 ^2^
Number of Doses (Interval):	2 doses (3 days ^1^)	3 doses (3–4 weeks ^3^)
Time to complete antibody release:	3–5 days ^1,2^	3–5 days
Administration window:	3–5 min	5–10 min

References: ^1^ [13,14], ^2^ [18], ^3^ [4].

## Data Availability

The raw data supporting the conclusions of this article will be made available by the authors upon request.

## Supplementary Materials

The following supporting information can be downloaded at: https://www.mdpi.com/article/10.3390/gels12060489/s1, Figure S1: Impact of antibody concentration on the hydrogel shear storage modulus G′ (Pa). Hydrogels were formulated with human IgG across a range of concentrations (1, 5, 15 mg/mL) in combination with hyaluronic acid (1.2% *w*/*v* final), PEG-DA (1.0% *w*/*v* final), rHuPH20 (1660 IU/mL final). The shear storage modulus (G′) was measured using an ElastoSens rheometer over a 60-min period at 1-min intervals after complete mixing of the component reagents. Replicate measurements for the 15 mg/mL dose of human IgG are included as an internal assessment for inter-test variability. Separate experiments assessing a 10 mg/mL IgG dose are presented in the Figure 1, main manuscript.

## Abbreviations

The following abbreviations are used in this manuscript:

AUC	area under the curve
Cryo-SEM	cryogenic scanning electron microscopy
cGMP	current good manufacturing practices
CTLA-4	cytotoxic T-lymphocyte-associated protein 4
G′	shear storage moduli
HA	hyaluronic acid
ICI	immune checkpoint inhibitor
irAEs	immune-related adverse event
Ig	immunoglobulin
IP	intraperitoneal
mAbs	monoclonal antibodies
NSG	non-obese diabetic, severe combined immune deficiency, interleukin-2 receptor common gamma chain-deficient
PK	pharmacokinetics
PEG-DA	poly-(ethylene glycol)-diacrylate
PD-1	programmed cell death protein 1
PD-L1	programmed death-ligand 1
rHuPH20	recombinant human hyaluronidase
TDLN	tumor-draining lymph node
Treg	regulatory T cell
