# Supplementary material for: Translating a Preclinical Hydrogel Platform into a Human Therapeutic for Delivering Targeted Low-Dose Anti-CTLA-4
Source: Gels. 2026 Jun 2;12(6):489. doi: 10.3390/gels12060489 (PMC13298415; doi:10.3390/gels12060489)
Supplement: Supplementary file 1 [file gels-12-00489-s001.zip › gels-4301933-supplementary.pdf]

Supplemental Figure S1 for:

Translating a Preclinical Hydrogel Platform into a Human Therapeutic for Delivering Targeted Low-Dose Anti-CTLA-4

Airi Harui [1] and Michael D. Roth[2,*]

[1] Division of Pulmonary & Critical Care, Department of Medicine, David Geffen School of Medicine at UCLA, Los Angeles, CA 90095-1690, USA; aharui@mednet.ucla.edu
[2] Division of Pulmonary & Critical Care, Department of Medicine, David Geffen School of Medicine at UCLA, Los Angeles, CA 90095-1690, USA; mroth@mednet.ucla.edu
* Correspondence: mroth@mednet.ucla.edu

Submitted for publication in *Gels* on April 20, 2026

Supplemental Figure S1:

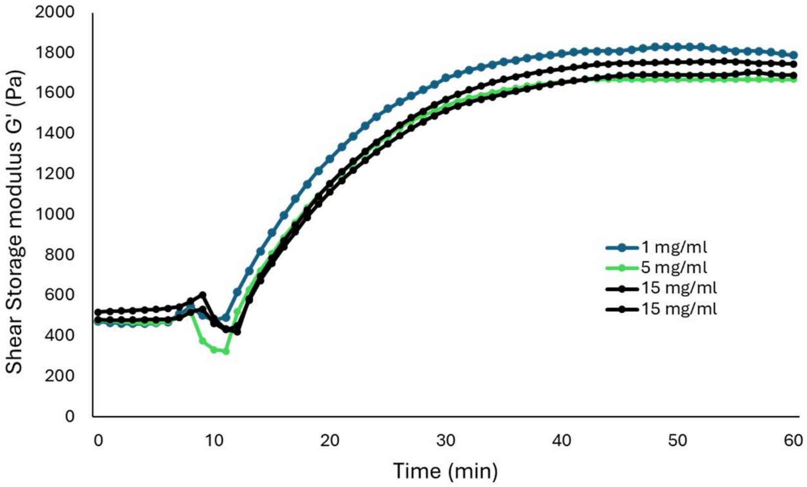

Figure S1. Impact of antibody concentration on the hydrogel shear storage modulus G′ (Pa). Hydrogels were formulated with human IgG across a range of concentrations (1, 5, 15 mg/ml) in combination with hyaluronic acid (1.2% w/v final), PEG-DA (1.0% w/v final), rHuPH20 (1660 IU/ml final). The shear storage modulus (G′) was measured using an ElastoSens rheometer over a 60-minute period at 1-minute intervals after complete mixing of the component reagents. Replicate measurements for the 15 mg/ml dose of human IgG are included as an internal assessment for inter-test variability. Separate experiments assessing a 10 mg/ml IgG dose are presented in the Figure 1, main manuscript.
